# Quercetin Reduces the Virulence of S. aureus by Targeting ClpP to Protect Mice from MRSA-Induced Lethal Pneumonia

**DOI:** 10.1128/spectrum.02340-21

**Published:** 2022-03-23

**Authors:** Shisong Jing, Xiangri Kong, Li Wang, Heming Wang, Jiaxuan Feng, Lin Wei, Ying Meng, Chang Liu, Xiren Chang, Yishen Qu, Jiyu Guan, Haimiao Yang, Chi Zhang, Yicheng Zhao, Wu Song

**Affiliations:** a Clinical Medical College, Changchun University of Chinese Medicine, Changchun, China; b Affiliated Hospital to Changchun University of Chinese Medicine, Changchun, China; c Department of Gastroenterology and Hepatology, Zhongshan Hospital Fudan University, Shanghai, China; d Department of Anesthesiology, Peking University Third Hospital, Beijing, China; e Key Laboratory of Zoonosis, Ministry of Education, College of Veterinary Medicine, Jilin University, Changchun, China; f School of Traditional Chinese Medicine, Changchun University of Chinese Medicine, Changchun, China; National Institutes of Health

**Keywords:** MRSA, antivirulence, inhibitor, caseinolytic peptidase P, pneumonia

## Abstract

The dramatic increase of methicillin-resistant Staphylococcus aureus (MRSA) poses a great challenge to the treatment of Staphylococcus aureus (S. aureus) infections. Therefore, there is an urgent need to identify novel anti-infective agents to attack new targets to overcome antibiotic resistance. Casein hydrolase P (ClpP) is a key virulence factor in S. aureus to maintain cellular homeostasis. We screened from flavonoids and finally determined that quercetin could effectively attenuate the virulence of MRSA. The results of the thermal shift assay showed that quercetin could bind to ClpP and reduce the thermal stability of ClpP, and the *K_D_* value between quercetin and ClpP was 197 nM as determined by localized surface plasmon resonance. We found that quercetin exhibited a protective role of a mouse model of MRSA-induced lethal infection in a murine model. Based on the above facts, quercetin, as a ClpP inhibitor, could be further developed as a potential candidate for antivirulence agents to combat S. aureus infections.

**IMPORTANCE** The resistance of Staphylococcus aureus (S. aureus) to various antibiotics has increased dramatically, and thus the development of new anti-infective drugs with new targets is urgently needed to combat resistance. Caseinolytic peptidase P (ClpP) is a casein hydrolase that has been shown to regulate a variety of important virulence factors in S. aureus. Here, we found that quercetin, a small-molecule compound from traditional Chinese herbal flavonoids, effectively inhibits ClpP activity. Quercetin attenuates the expression of multiple virulence factors in S. aureus and effectively protects mice from lethal pneumonia caused by MRSA. In conclusion, we determined that quercetin is a ClpP inhibitor and an effective lead compound for the development of a virulence factor-based treatment for S. aureus infection.

## INTRODUCTION

The emergence of antibiotic resistance in many opportunistic bacteria and their spread across species has led to the emergence of multidrug resistant pathogens (1). Staphylococcus aureus (S. aureus), in particular, not only has the ability to develop antibiotic resistance rapidly, but also to spread antibiotic resistance (2). Methicillin-resistant Staphylococcus aureus (MRSA) has spread rapidly in recent years and this type of S. aureus is resistant to all known *β*-lactam antibiotics, making the treatment of S. aureus infections increasingly challenging and posing a great threat to human health (3).

Targeting the virulence of S. aureus is a novel strategy that has been proposed (4). Virulence factors play a crucial role in the infection and invasion of S. aureus to the host, but have little impact on the *in vitro* survival of the bacteria (5). Antivirulence strategies do not exert as intense selective pressure on bacteria as antibiotics and avoid adverse negative effects on typical microorganisms (6). Multiple global transcription factors synergistically regulate the expression of virulence factors in S. aureus, so the effect on a single virulence factor is often unsatisfactory. It has been shown that immunization with antibodies to four important surface-associated factors in S. aureus in combination produces significant protective immunity (7). It is possible to interfere with several or more virulence expression through small molecule compounds that effectively control infections brought about by S. aureus.

The casein hydrolase ClpP is widely present in various organisms, and its most important function is to maintain intracellular protein homeostasis (8). The hydrolytic ability of ClpP protease is essential for the pathogenicity of S. aureus. ClpP is involved in the regulation of accessory gene regulator (*agr*) and other regulatory pathways (MgrA and SarA) and may also affect the expression of a variety of extracellular virulence and anchoring proteins. For example, the extracellular protein hemolysin (hla), is significantly reduced in *clpP* knockout S. aureus (9). It is worth mentioning that ClpP is also involved in the initiation of biofilm adhesion and could influence the formation of biofilms (10). The pathogenicity of *clpP* deletion mutant strains of S. aureus was significantly reduced (9). According to the above analysis, virulence factors regulated by ClpP are involved in almost every process of bacterial infection of the host. ClpP represents a highly promising antimicrobial target, which has a significant regulatory effect on the virulence of multiple pathogenic bacteria.

Recently, we identified a flavonoid, quercetin, from an existing library of natural herbal monomeric small molecules that could effectively inhibit the activity of ClpP and could cut down the virulence of MRSA. Quercetin is a five-hydroxyl antioxidant flavonoid with anti-inflammatory and anti-cancer properties (11, 12). Herein, we found that quercetin could inhibit the expression of important virulence factors such as hla and pvl without affecting the growth of S. aureus, effectively weaken the pathogenicity of S. aureus. Further results of cellular thermal shift assay (CETSA) and localized surface plasmon resonance (LSPR) revealed a strong binding between quercetin and ClpP, and the binding mode between the two was simulated by molecular dynamics. The final animal results indicated that quercetin could protect mice from lethal pneumonia brought on by MRSA. These results demonstrated the mechanism of quercetin inhibiting the virulence of S. aureus and provided a theoretical basis for the further development of quercetin as an adjuvant therapeutic agent in combating S. aureus infections.

## RESULTS

### Quercetin is an inhibitor of ClpP.

**FIG 1:**
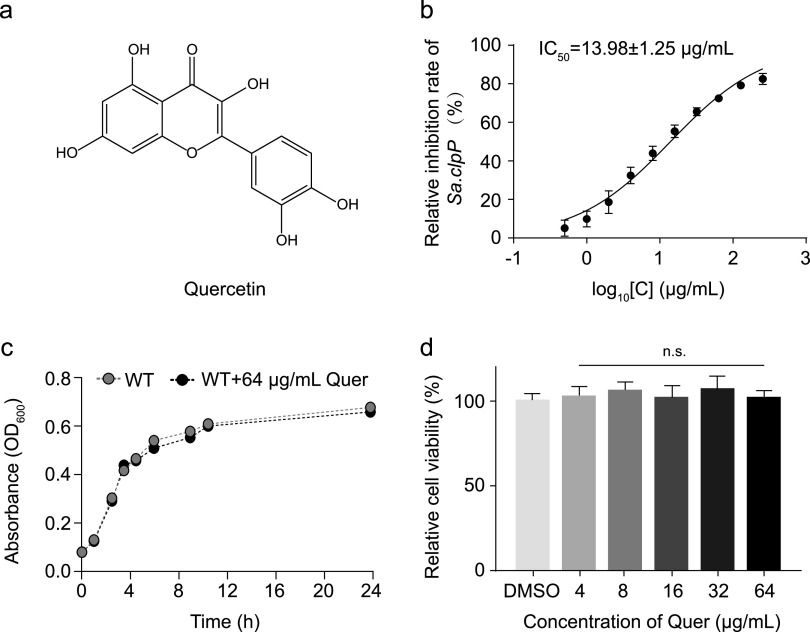
Quercetin was found to be an inhibitor of ClpP from natural products. (a) Structure of quercetin. (b) The IC_50_ value of quercetin on ClpP was (13.98 ± 1.25 μg/mL) based on the fluorescent substrate Suc-LY-AMC. (c) The growth of USA300 is unaffected by 64 μg/mL quercetin. Wild type USA300 was used as a control. (d) Quercetin at 64 μg/mL has no effect on the viability of Vero cells.

Natural products could inhibit bacterial virulence and defend against various pathogens through different pathways of action and are also an essential source for the design of new agents (13, 14). Since the fluorescent substrate Suc-LY-AMC can be used to detect the activity of ClpP protein, we screened a library of natural products available in our laboratory. There are 459 compounds in the natural product library, which are mainly composed of flavonoids, terpenoids, alkaloids and quinones. Three primary positive compounds were identified from the primary screening, with a hit rate of 0.65%. Since all candidate compounds belong to flavonoids, we placed the details of the 109 flavonoids in the Table S3. The MIC of the three candidate compounds was further determined and it was found that nor-kurarinone and isobavachalcone exhibited strong bacterial inhibition at very low concentrations, so quercetin was finally selected for the next experiments (Table S3). Quercetin could effectively inhibit the activity of ClpP with IC_50_ value of 13.98 ± 1.25 μg/mL (Fig. 1a and b). Then we determined the MIC of quercetin against USA300 as 128 μg/mL. The growth of S. aureus USA300 in the presence of quercetin were further determined and the results indicated that 64 μg/mL of quercetin had no inhibitory effect on the growth of S. aureus USA300 and was less likely to develop antibiotic resistance to S. aureus USA300. To further investigate the safety of quercetin, we examined the cytotoxicity of quercetin on eukaryotic cells and the results showed that quercetin did not affect the proliferation of Vero cells at a 64 μg/mL (Fig. 1d).

### Quercetin significantly reduces the virulence of S. aureus *in vitro*.

**FIG 2:**
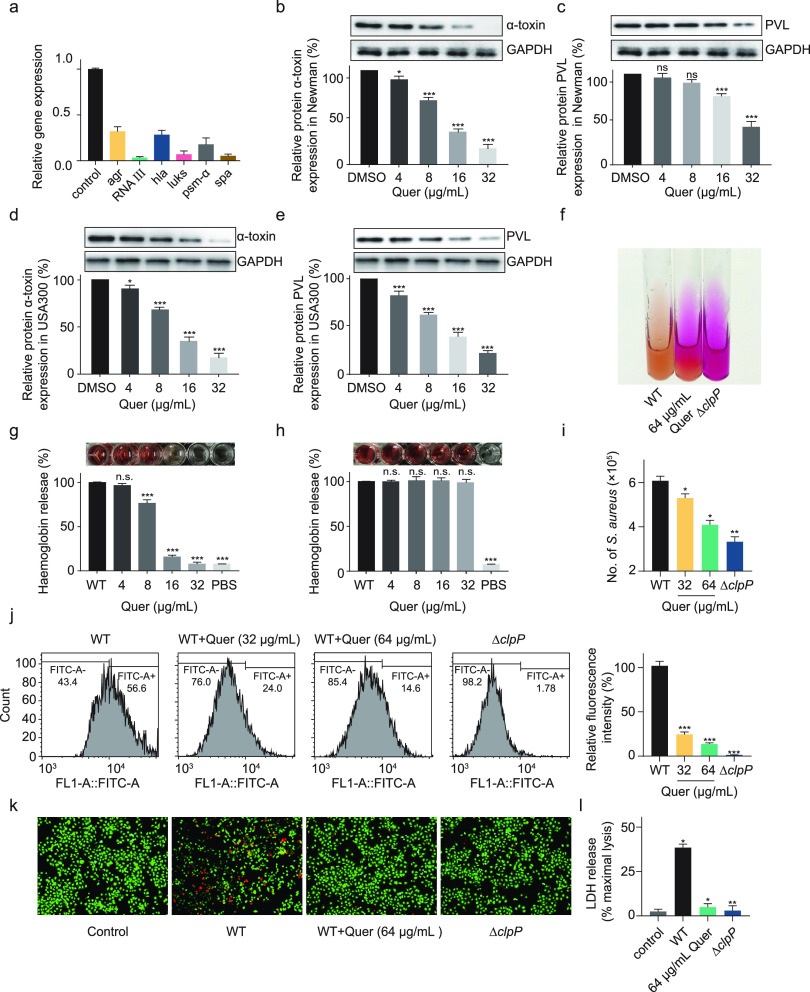
Quercetin significantly reduces the virulence of S. aureus *in vitro*. (a) Expression levels of *agr*, *RNAIII*, *hla*, *luks*, *psm-α* and *spa* were determined by qPCR in the presence of 32 μg/mL of quercetin. (b-e) Quantification of alpha-toxin and PVL expression levels in S. aureus USA300 and Newman under the effect of different concentrations of quercetin by Western blotting, and their corresponding gray value analysis. (f) Urease production in USA300 was induced by quercetin. Δ*clpP* served as a positive control. (g and h) The effect of different concentrations of quercetin on the hemolytic capacity of S. aureus USA300 and USA300 supernatant. (i) Effect of quercetin on the ability of S. aureus to invade A549 cells. A549 cells were lysed, and intracellular bacteria were determined by plate counting. (j) Flow cytometry determination of FITC-labeled SpA on the surface of S. aureus and their corresponding relative fluorescence intensity. Δ*clpP* served as a positive control. (k) Fluorescence microscope image of mouse macrophages J774 cells stained with live/dead assay. The green and red fluorescence indicated live and dead cells, respectively. (l) LDH release is presented as % of total LDH. Significance is calculated based on one-way ANOVA: **, *P* < 0.01 and ***, *P* < 0.001.

Virulence factors can aid invasion and colonization of S. aureus into the host and aid immune evasion by S. aureus, which is critical for controlling S. aureus infections. ClpP plays a crucial role in the production of virulence factors in S. aureus, therefore, we examined the effect of 32 μg/mL of quercetin on the transcript levels of important virulence gene such as *RNAIII*, *hla*, *luks*, *psm-α* and *spa*. The Agr population sensing system could initiate the expression of several virulence factors through RNAIII, both of which showed about 3-fold and 50-fold decreases in transcript levels, respectively. The important virulence factors *hla*, *luks*, *psm-α* and *spa* in were significantly downregulated in S. aureus, especially *lukSF*, which is responsible for the encoding of leukocidin (PVL), decreased approximately 10-fold (Fig. 2a). The hemolysin (alpha-toxin) and leukocidin (PVL) could kill a variety of cells in the host and play an essential role in the cure of S. aureus (15). We examined the production of alpha-toxin and PVL in USA300 and Newman in the presence of quercetin at different concentrations and found that quercetin inhibited alpha-toxin and PVL in a concentration-dependent manner (Fig. 2b to e).

Urease is not only a vital component of the acidic stress response system of S. aureus, but also a crucial factor in the pathogenesis of S. aureus (16). Taking advantage of the characteristic that urease agar basal medium changes color in the presence of urease, we examined the production of urease in S. aureus in the presence of quercetin. The results were shown in Fig. 2f, *clpP* deficiency in S. aureus causes the medium to become completely red, while the addition of quercetin causes almost the same effect, indicating that quercetin is able to induce an increase in urease in S. aureus and disrupt the intracellular pH homeostasis induced by urease. On the other hand, this result also suggested that the effect of quercetin on urease may be achieved through ClpP. Next, we examined the mode of inhibition of hemolysin by quercetin through defibrinated rabbit blood (17). The results are shown in Fig. 2g, that coculture of quercetin with S. aureus was able to effectively inhibit the hemolysis of S. aureus in a concentration-dependent manner. However, the coincubation of quercetin with the supernatant of S. aureus cultures did not inhibit the hemolysis. The results suggest that quercetin inhibits the hemolytic ability of S. aureus by inhibiting the expression rather than the activity of alpha-toxin (Fig. 2h).

S. aureus usually colonizes the nasopharynx of humans asymptomatically and metastasizes to lung tissue causing pneumonia. We determined the invasive ability of S. aureus in the presence of quercetin by human lung A549 cells. It was found that quercetin could suppress the invasive ability of S. aureus in a dose-dependent manner and thus preventing the invasion of A549 cells by S. aureus (Fig. 2i). Protein A (SpA) is a very critical virulence factor for S. aureus and is considered to be an essential component of the immune evasion mechanism of this pathogen (18). Since SpA can specifically bind IgG with Fc fragment, the content of S. aureus surface protein SpA can be assessed by flow cytometry detection of fluorescence intensity of FITC-labeled IgG. The fluorescence intensity of S. aureus treated with different concentrations of quercetin was found to be significantly diminished, revealing that quercetin reduces the anchoring of surface protein SpA to the cell wall. (Fig. 2j). Another way for S. aureus to “immune evade” is to kill leukocytes in the host. PVL (LukF-PV at 34 kDa/LukS-PV at 33 kDa) showed cytolytic activity against leukocytes and was highly cell-specific (19). We examined the killing ability of S. aureus on mouse macrophages J774 in the presence of quercetin by live/dead assay (green: live, red: dead) and found that quercetin was effective in preventing S. aureus from killing macrophages (Fig. 2k). The lactate dehydrogenase (LDH) assay could likewise be used to detect the death of J774 cells. Quercetin could effectively protect cells from S. aureus and reduce cell mortality (Fig. 2l). The results of these analyses showed that quercetin could reduce the levels of several important virulence factors of S. aureus and suggested that this may be achieved through ClpP.

### Quercetin can bind to ClpP.

**FIG 3:**
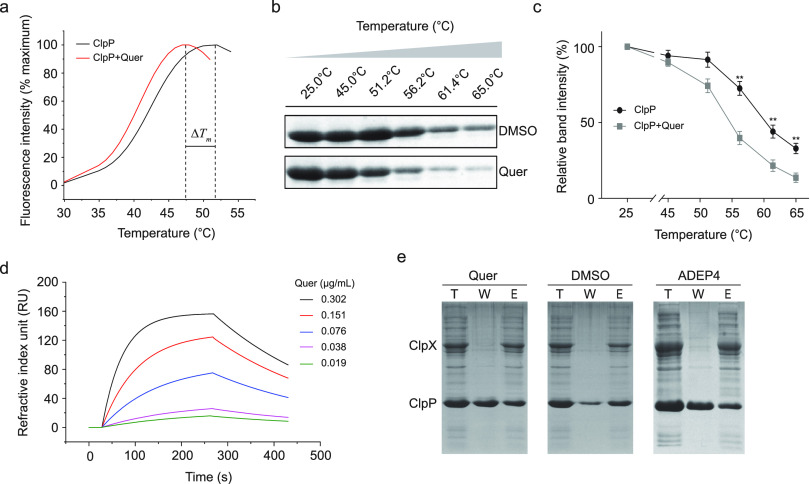
Quercetin can bind to ClpP. (a) The binding between quercetin and ClpP was examined by TSA. Quercetin caused a decrease in the thermal stability of ClpP. (b and c) SDS-PAGE analysis (b) and thermal shift assay curves (c) showing that quercetin caused a decrease in the *T_m_* of ClpP protein in the cell lysates. The complete gel image is shown in Fig. S1. (d) Surface plasmon resonance imaging reveals the kinetics of quercetin binding to ClpP. The chromaticity lines represent the response of the dynamical signal. (e) T (Total) indicates the amount of protein initially added to ClpP and ClpX. W (Wash) indicates the effluent from the ClpXP complex after flowing through the Ni-NTA and eluting with a binding buffer without imidazole. E (Elute) indicates the effluent eluted with a binding buffer containing high concentration of imidazole. Quercetin could bind ClpP to dissociate ClpP from the ClpX complex. ADEP4 served as a positive control. Significance is calculated based on two-tailed *t* test: **, *P* < 0.01 and ***, *P* < 0.001.

Binding is a prerequisite for natural compounds to interact with proteins. Thermal shift assay (TSA), also known as differential scanning fluorimetry (DSF), has become an important label-free technique for biophysical ligand screening and protein engineering (20). TSA is able to detect changes in the thermal stability of the target protein after binding small molecules to determine the interaction between them (20). As shown in Fig. 3a, the *T_m_* value of ClpP decreased by more than 2°C with the addition of quercetin, indicating that quercetin was able to bind to ClpP and reduce the thermal stability of ClpP. Since the binding of natural compounds and target proteins occurs in a more complex environment, we examined the interaction between quercetin and ClpP proteins by cellular thermal shift assays (CETSA). CETSA provides a label free biophysical method that facilitates the direct measurement of cellular target engagement (21). The results showed that in Fig. 3b (SDS-PAGE images) and 3c (grayscale analysis), ClpP became unstable and showed a decrease as temperature increases. With the addition of quercetin, ClpP became more unstable, indicating that there was a binding between quercetin and ClpP. Localized surface plasmon resonance (LSPR) is a collective oscillation of conduction band free electrons in metallic nanostructures due to their interactions with light (22). LSPR is able to respond to kinetic processes such as binding, dissociation, and molecular recognition between drug molecules and target proteins in their natural state (23). We experimentally verified the binding behavior of quercetin to ClpP in the LSPR analysis (Fig. 3d). The association rate constant *K_a_* and the dissociation rate constant *K_d_* were 1.88 × 10^4^ M^−1^s^−1^ and 3.70 × 10^−3^ s^−1^, respectively, in the ligand- within the hierarchy of protein interactions (24). The equilibrium dissociation constant (*K_D_* = 1.97 × 10^−7^ M) indicates that ClpP and quercetin have a very powerful binding capacity. ClpP exerts a hydrolytic function by forming a ClpXP complex with ClpX under normal physiological conditions. Therefore, we expressed purified 6×His-ClpX protein and excised the His tag on ClpP, when the mixture containing these two proteins passed through Ni-NTA, ClpP was able to bind to 6×His-ClpX without efflux. We added quercetin to the mixture and observed whether quercetin could bind ClpP and drag ClpP down. The results showed that quercetin was able to separate from Ni-NTA, indicating that quercetin interferes with the binding of ClpP to ClpX (Fig. 3e). In summary, in combination with the above results, a significant direct interaction between quercetin and ClpP was confirmed.

### Molecular modeling (MD) and mutagenesis study.

**FIG 4:**
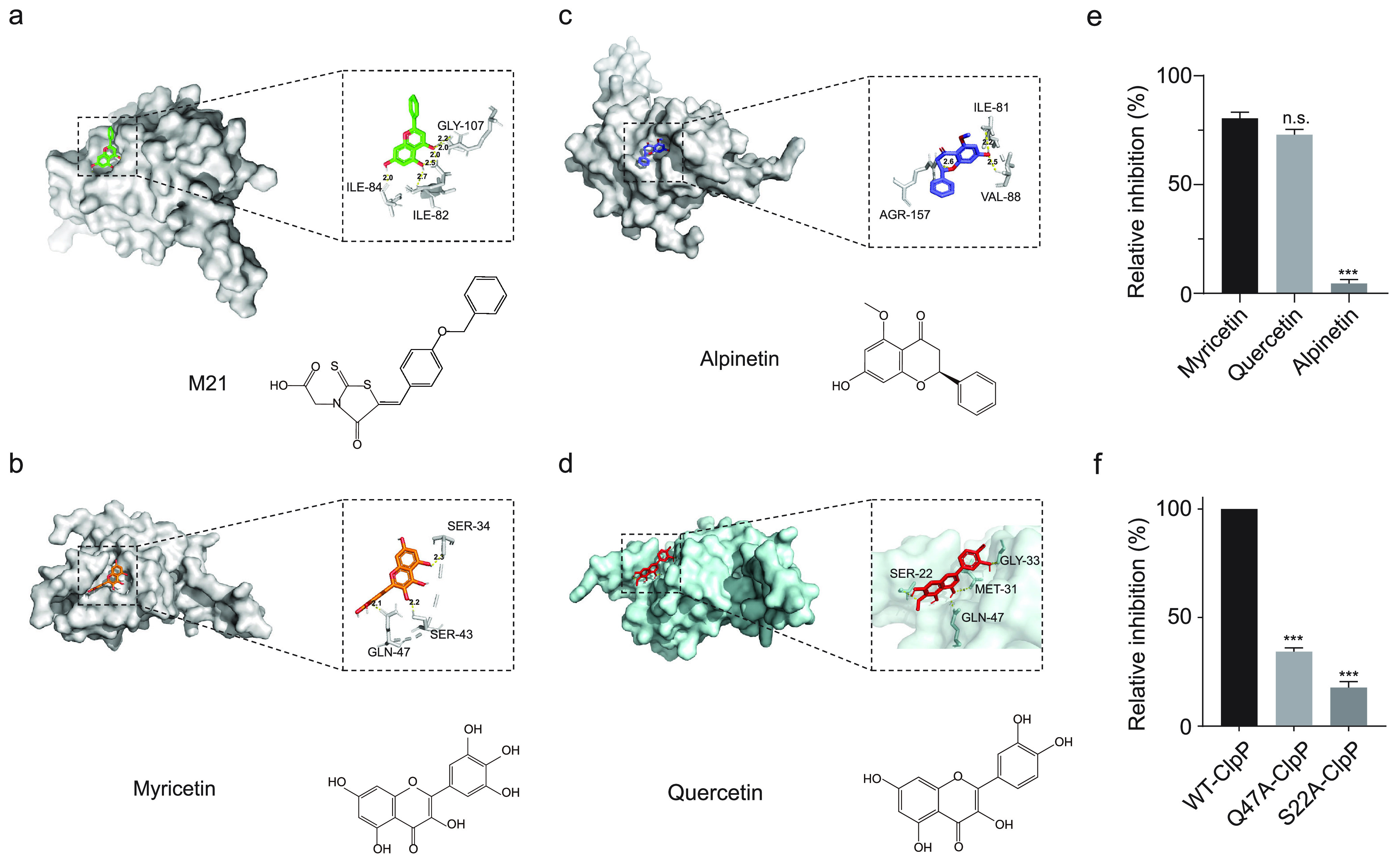
Molecular modeling of ClpP-quercetin binding. (a-d) The results of the predicted molecular docking of M21, myricetin, alpinetin and quercetin with ClpP protein and the structural formulae of each compound, respectively. M21 and myricetin were used as positive controls. (e) The inhibition of ClpP activity by structurally similar flavonoids was determined by Suc-LY-AMC. Myricetin was used as positive control. (f) Two mutants Q47A-ClpP and S22A-ClpP were resistance to quercetin inhibition. Significance is calculated based on two-tailed *t* test: *, *P* < 0.05 and ***, *P* < 0.001.

Molecular docking is able to demonstrate the details of small molecule drug binding to target proteins at the molecular level and has become an essential tool for studying the relationship between biomolecules and small molecule drugs (25). We performed molecular docking of several small molecules and ClpP proteins, among which M21 and myricetin, which have been reported as ClpP inhibitors, were used as positive controls (Fig. 4a to d) (26, 27). Myricetin, quercetin and alpinetin are simple and structurally similar flavonoids, but their molecular docking results differed considerably. The docking score of quercetin was −6.7, which was very close to the positive-control M21 and popcornin (docking score: −6.9), and differed significantly from alpinetin (docking score: −5.9), indicating a large difference in the affinity of structurally similar natural compounds for ClpP. We further determined the inhibitory ability of these flavonoids on ClpP activity by using Suc-LY-AMC, a specific fluorescent substrate for ClpP. As shown in Fig. 4e, the inhibitory ability of quercetin and the positive-control myricetin on ClpP activity were the closest, but the inhibitory ability of dihydromyricetin and chrysin had less than 50% inhibitory ability on ClpP activity. This result implied that there is structural specificity in the inhibition of ClpP activity by quercetin. The results of molecular docking of quercetin and ClpP were illustrated in Fig. 4d, residue Met-31 is very close to quercetin, forming a strong hydrogen bonding interaction. Further study revealed that residues Gly-33 and Gln-47 could form two hydrogen bonds with two hydroxyl groups of quercetin with solid hydrogen bonding interactions, which is essential for quercetin binding to quercetin ClpP. Since ClpP is a complex structure, it needs to bind to the chaperone protein ClpX to form the ClpXP complex to perform its function. Quercetin is located near the ClpP binding pocket in molecular docking and may exert its inhibitory effect by preventing ClpP from forming the ClpXP complex. Based on the results of molecular docking, mutant proteins G33A-ClpP and Q47A-ClpP were constructed and expressed. Both mutants efficiently cleaved the fluorescent substrate Suc-LY-AMC, but both were resistant to quercetin inhibition to some extent (Fig. 4f). Thus, amino acid residues Gly-33 and Gln-47 are involved in the binding process of quercetin to ClpP, which is consistent with the results of MD simulations.

### Quercetin can protect mice from lethal MRSA infection.

**FIG 5:**
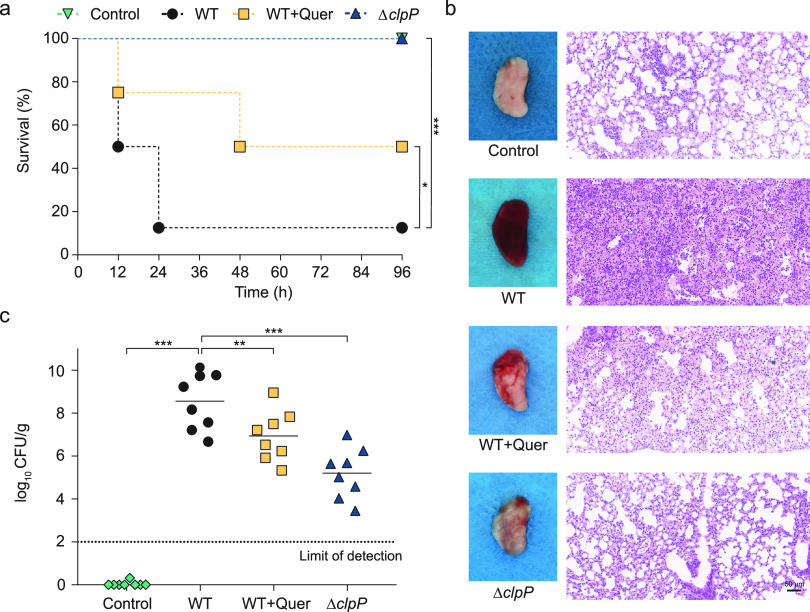
Quercetin protects mice from MRSA pneumonia. (a) Survival of mice treated with quercetin (100 mg/kg) at the indicated times after infection with WT (USA300, 2e8 CFU/30 μL). Significance (*P* value) in the panels except (a) is calculated using log-rank test: **, *P* < 0.01 and ***, *P* < 0.001. (b) Gross and histopathology of S. aureus WT and WT-Δ*clpP* infected lung tissue from mice. Quercetin (100 mg/kg) treatment by subcutaneous injection. Scale bar, 50 μm. (c) The infectious bacterial load in the lung of mice with quercetin (100 mg/kg) treatment. In the graph, horizontal bars indicate the mean of bacterial load measurements, each dot represents a mouse. Significance is calculated based on one-way ANOVA: **, *P* < 0.01 and ***, *P* < 0.001.

S. aureus cause severe pulmonary inflammation, leading to high morbidity and mortality (28, 29). To evaluate the contributions of quercetin to resistance to MRSA infection, mice were intranasally infected with USA300 using a mouse intranasal infection model (pneumonia model). Mice were inoculated with 2 × 10^8^ CFU of S. aureus and treated by subcutaneous injection of 100 mg/kg of quercetin. Consistent with previous studies (30), mice were not able to resist the attack of S. aureus, with a survival rate of only 20% over 96 h. In contrast, the ability of S. aureus to infect mice was greatly reduced in the absence of *clpP*, and all mice survived (Fig. 5a), suggesting an important regulatory role of ClpP for the process of S. aureus infection of the lungs. After treatment with quercetin, the survival rate of mice increased to 50%, indicating that quercetin has a protective effect on the lung tissue of mice infected with S. aureus.

Next, we investigated the damage to the lung tissue of mice with S. aureus pneumonia, as shown in Fig. 5b, the lung tissue of the control group was light pink, soft and elastic, and the alveolar cavity was full, with no infection. The lung tissues of the *clpP*-deficient S. aureus-infected mice had a small amount of congestion, which was generally consistent with the control group. Whereas the lungs infected with S. aureus were dark red, hard in texture, with loss of elasticity, severe congestion of the alveolar cavity, and the presence of an extensive infiltration of inflammatory cells. After quercetin treatment, the lung tissue congestion was improved, elasticity was restored, and inflammatory cell infiltration in the alveolar cavity was reduced, all of which indicated that quercetin had a protective effect on the lungs of mice. The lung bacterial load of mice in the quercetin-treated group decreased from 8.72 × 10^8^ CFU to 7.45 × 10^6^ CFU compared with the infected group, which was consistent with H&E staining results (Fig. 5c). In conclusion, these results suggested that quercetin renders MRSA less pathogenic in this mouse infection model and the protected mice from lethal infection with MRSA.

## DISCUSSION

The misuse of antibiotics has led to the emergence and spread of MRSA, making the infections caused by S. aureus more and more challenging to treat. The pathogenicity of S. aureus is strongly dependent on the virulence factor, and the virulence against the bacteria minimizes the development of bacterial resistance and does not harm the normal microflora of the organism. Hence, the anti-virulence strategy is very promising (6, 31). The expression of virulence factors is a very complex process, regulated by multiple regulatory factors. Inhibition of one virulence factor alone can no longer achieve good therapeutic effects, and simultaneous inhibition of multiple virulence factors is an important research direction in the anti-virulence strategy. ClpP is able to regulate several important virulence factors in S. aureus, which are involved in almost every process of bacterial infection of the host, and therefore ClpP is of interest as a novel anti-virulence target.

Natural products are essential medicinal resources and also a source of novel anti-virulence drugs and antibiotics, and several natural products have been identified as having good antibacterial activity and anti-virulence effects (14, 32). Therefore, we screened inhibitors of ClpP from our existing natural product library and found that quercetin was able to effectively inhibit the activity of ClpP. Our findings demonstrated that the expression of almost all important virulence factors in S. aureus was attenuated by ClpP inhibition. For instance, the cellular pH homeostasis, the ability to invade host cells, the capacity to kill erythrocytes and leukocytes cell, and immune evasion were significantly reduced, ultimately manifesting as a severe weakening of S. aureus pathogenicity.

Since the binding of ligands to target proteins is frequently found in more complex environments, CETSA is able to further validate the binding between small molecule drugs and target proteins. Our results showed that quercetin is able to bind ClpP and reduce its thermal stability. In the following LSPR results, we determined a *K_D_* value of 1.97 × 10^−7^ M for quercetin and ClpP, indicating an unparalleled binding capacity between the two. We further simulated molecular docking for the ClpP protein and quercetin in order to understand their binding traits. The oligomeric state of ClpP is a hollow barrel structure consisting of seven ClpP monomers. Fourteen ClpP protomers arrange themselves to form a tetradecameric barrel-shaped complex in a stack of two heptameric rings. The active center of ClpP is located inside ClpP to facilitate the hydrolytic effect. Under normal physiological conditions, ClpP is required to bind to the chaperone protein ClpX (in S. aureus) to form the ClpXP complex for its function. The results of molecular docking showed that although quercetin did not bind to the key active site of ClpP (Ser-98), we found that S22, M31, G33 and Q47, where quercetin binds ClpP, are near the interface where ClpP binds to ClpX. We found that quercetin could indeed affect the binding of ClpP to ClpX by pulldown experiments, suggesting that quercetin may affect the virulence level of S. aureus by preventing the formation of the ClpXP complex and causing ClpP to fail to perform its normal function. Meanwhile, we found by FRET experiments and molecular docking that not all small flavonoid compounds with similar structures could inhibit the ClpP production activity, suggesting that the inhibition of ClpP by quercetin is structure-specific.

ClpP is an essential protein that regulates homeostasis and stress in S. aureus and is critical for the developing pneumonia in S. aureus infections (33). This is corroborated by our results that quercetin was able to attenuate the virulence of S. aureus in mice by inhibiting ClpP thereby reducing lung damage. Quercetin is a natural flavonoid herbal monomer compound that has been shown to exert antimicrobial effects in combination with other small molecule drugs or antibiotics that could enhance the susceptibility of MRSA to antibiotics (34, 35). ClpP is able to manage the stress of S. aureus to the outside world thereby regulating the susceptibility of S. aureus to antibiotics. Therefore, we speculate that the inhibition of ClpP activity by quercetin may be one of the reasons for altering the susceptibility of S. aureus to antibiotics. Since quercetin has a very superior anti-virulence activity and is able to overcome the problem of antibiotic resistance in pathogenic bacteria, quercetin could be further developed as a promising antivirulence agent.

## MATERIALS AND METHODS

### Bacterial strains, plasmids, culture coditions, and reagents.

The bacterial strains and plasmids used in this study are listed in Table S1. Newman as a laboratory standard for evaluating drug efficacy and USA300 as a very virulent MRSA were used in this study, respectively. Unless otherwise stated, S. aureus USA300 was used in all experiments, and in addition, all cultures were incubated at 37°C, 180 rpm/min. *E. coil* strains were generally cultured in Luria Bertani (LB, Hopebio, Qingdao, China) broth or LB agar plates, and S. aureus was generally grown in Tryptice Soy Broth (TSB, Hopebio, Qingdao, China) or Tryptice Soy Agar (TSA). Fifty μg/mL of kanamycin and 10 μg/mL of chloramphenicol were used for plasmid selection in *E. coil* and S. aureus, respectively. Quercetin (Cat number JOT-10049, Chengdu Pufei De Biotech Co., Ltd., 98% purity) was dissolved in DMSO as 10 mg/mL mother liquor.

### MIC assay and growth assay.

The MIC was determined based on the broth microdilution method in the CLSI guidelines (36). Briefly, 5 × 10^4^ S. aureus cells were inoculated in 100 μL of CAMHB medium in a 96-well plate, followed by the addition of serial dilutions of quercetin, and MIC was determined after incubation at 37°C for 18 h. To further investigate the inhibitory effect of quercetin on the growth of S. aureus, USA300 was inoculated into TSB medium containing 64 μg/mL quercetin, and the absorbance values of the cultures at OD_600_ were measured over 24 h.

### Cytotoxicity assay.

The cytotoxicity of quercetin toward Vero cells (African green monkey kidney cell line) was determined by MTT according to the manufacturer’s instructions. Briefly, Vero cells (8 × 10^3^ cells/well) were seeded into 96-well plates for culture. After 24 h, they were treated with 100 μL of drug-containing medium. Afterwards, the medium was replaced with 100 μL of MTT solution (final concentration 0.5 mg/mL) and incubated at 37°C for 4 h. The MTT solution was removed, MTT formazan was dissolved with 100 μL of DMSO, and the optical density (OD) values at 490 nm were measured.

### Protein expression and purification.

Protein expression was performed in E. coli strain BL21 (DE3) with the following procedure: BL21 strain containing plasmid pET28a-clpP was inoculated in LB broth containing 50 μg/mL of kanamycin. When the absorbance value at 600 nm was about 0.6, 0.5 mM isopropyl-β-d-thiogalactoside (IPTG) was added and induced overnight at 20°C with shaking (160 r.p.m.). Cells were harvested, resuspended in lysis buffer (40 mM Tris, pH = 8.5), and lysed by sonication at low temperature. The lysate was cleared by centrifugation at 18,000g for 1 h at 4°C, then the supernatant was applied to His-Trap column (GE Healthcare). The ClpP protein was eluted by gradient imidazole solution and verified by SDS-PAGE. The mutant proteins were expressed and purified following the same protocol as that for the wild-type protein.

### Screening of ClpP inhibitors.

ClpP has hydrolytic substrate activity when present alone, but at this time, the substrate can only be some short peptides such as Suc-LeuTyr-AMC (37). This property was exploited to screen the available laboratory library of small molecules for ClpP inhibitors. First, 10 μM ClpP and 64 μg/mL of the small molecule compound were added to a black opaque 96-well plate containing 100 μL of ClpP buffer (100 mM HEPES,100 mM NaCl) and incubated for 1 h at room temperature. Afterward, 100 μM Suc-LeuTyr-AMC (Cat number S1153, Sigma-Aldrich, St. Louis, MO, USA) was added to 96-well plates and incubated for 30 min at 32°C. Finally, the fluorescence values were detected by Infinite M200 (360 nm excitation/465 nm emission).

### qPCR.

S. aureus was inoculated into TSB medium and treated with quercetin at a final concentration of 32 μg/mL added to the culture when the OD_600_ reached 0.3. Afterward, 5 × 10^8^ CFU of bacteria were collected and total RNA was extracted by MiniBEST Universal RNA Extraction kit (Cat number 9767, TaKaRa, Dalian, China) and the purity was identified using agarose electrophoresis. PrimeScript RT reagent kit (Cat number RR047Q, TaKaRa) was used for cDNA synthesis, ABI 7900HT real-time PCR system was used for qPCR analysis. The relative quantification of S. aureus transcripts was determined by the expression ratio of target transcripts relative to 16s (housekeeping genes). The experiments were performed three times independently. The primers used for qPCR are listed in Table S2.

### Western blot analysis.

After 12% SDS-PAGE separation of the samples, the proteins were transferred to PVDF (Millipore). PVDF membranes were then incubated with blocking buffer for 2 h. The rabbit anti-alpha-toxin polyclonal antibody (diluted 1:10,000) (Cat number S7531, Sigma-Aldrich) and the rabbit polyclonal anti-PVL LukS subunit (0.5 μg/mL, Cat number ab190473, Abcam, Cambridge, United Kingdom) were used to detect alpha-toxin and PVL, respectively. The HRP-labeled goat anti-rabbit IgG (diluted 1:2,000) (Cat number SE134, Solarbio, Beijing, China) was used as the secondary antibody. Western blotting bands were quantified using *Image J* Software.

### Hemolysis assay.

S. aureus was cultured in the TSB medium in the absence or presence of quercetin (4, 8, 16 and 32 μg/mL) or an equal volume of DMSO. When OD_600_ was 2.5, the supernatant of the culture was collected by centrifugation (5,500 g, 4°C, 3 min) and filtered through a 0.22 μm membrane to remove impurities. Next, 100 μL of supernatant, 875 μL of PBS and 25 μL of defibrinated rabbit blood were added to a 1.5 mL EP tube and incubated at 37°C for 30 min. Afterward, the samples were centrifuged and the absorbance values at 600 nm were measured. In addition, PBS without supernatant was used as a positive-control group.

### Urease activity.

Urease production was determined by using the urease agar base medium (Catnumber HB4095, Hopebio, Qingdao, China) with phenol red indicator. Urea in the medium is hydrolyzed enzymatically by urease to yield ammonia, thus turning the medium red. S. aureus was inoculated into urease agar base medium containing quercetin and the color change of the medium was observed after 2 days.

### Invasion assay.

Invasion of human lung A549 cells by S. aureus were performed as previously described (38). A549 cells were grown to confluence in 24-well plates. S. aureus was grown in medium with or without quercetin to an OD_600_ of 1.0, and the bacterial suspension was added to the 24-well plates and incubated for 1 h. Afterwards, the bacterial suspension was removed and washed three times with PBS. Fresh medium containing 300 μg gentamicin/mL was added to each well and incubated for 2 h to kill all extracellular bacteria. After washing three times again with PBS, cells were treated with 100 μL of 0.25% trypsin-EDTA and lysed by the addition of 400 μL of ice-cold 0.025% Triton X-100. Subsequently, appropriate dilutions were plated on TSB agar to determine the numbers of viable bacteria.

### Flow cytometry.

To quantify the effect of quercetin on SpA anchoring in S. aureus, S. aureus was labeled with FITC-IgG and measured by flow cytometry. In brief, S. aureus was grown in medium with or without quercetin to an OD_600_ of 1.0, and the bacteria were collected by centrifugation and washed by PBS. Subsequently, the bacteria were incubated in PBS solution containing 0.2% BSA (wt/vol) for 10 min. Bacteria were washed three times by PBS, resuspended by PBS containing a 1:50 dilution of FITC-labeled rabbit anti-goat-IgG (Cat number AP106F, Sigma-Aldrich), and incubated for 1 h in dark. After washing the bacteria adequately, they were fixed with 2% formaldehyde for 10 min, after which the fluorescent signal was detected on Flow cytometry (CytoFLEX, Beckman Coulter, Inc.) and analyzed using Flowjo software (FlowJo, LLC, v7.6.3).

### Live/dead and LDH release assay.

Mouse macrophage J774 was inoculated into 96-well plates at a density of approximately 20,000 cells per well. The cells were then cocultured with 200 μL of S. aureus at 37°C for 5 h. The cells were washed by PBS and stained with live/dead (green/red) reagent (Cat number 04511-1KT-F, Sigma-Aldrich), and the results were observed under a fluorescence microscope. The cytotoxicity assay kit (Cat number 11644793001, Roche, Basel, Switzerland) was used to assess the release of LDH from the supernatant in the wells.

### Thermal shift assay (TSA).

TSA was essentially done as described previously (20), TSA experiments were performed using the *Bio-Rad iQ5* (Bio-Rad). ClpP (final concentration, 2 μM) and TSA Buffer (150 mM NaCl,10 mM HEPES, pH = 7.5) were added to an opaque 96-well plate in the presence of SYPRO Orange (Cat number S5692, Sigma-Aldrich) transparent 96-well plates. The 96-well plates were heated from 25°C to 90°C at a rate of 1°C/min and the fluorescence of SYPRO Orange (Excitation: 475 nm, Emission: 590 nm) was detected.

### Cellular thermal shift assay (CETSA).

ClpP proteins were expressed using the same method, and the supernatant of the lysate was collected by centrifugation after ultrasonic fragmentation. The supernatant solution was centrifuged after ultrasonic lysis and incubated with 64 μg/mL of quercetin and an equal volume of DMSO with the supernatant solution for 1 h at room temperature. The mixed solution was heated at the indicated temperature for 5 min followed by an immediate ice bath, after which the supernatant solution was centrifuged to remove insoluble material and taken for SDS-PAGE analysis. SDS-PAGE gels were stained with Coomassie brilliant blue G-250 and the relative intensities of the target proteins were analyzed by Image J.

### Localized surface plasmon resonance (LSPR).

Interactions between and ClpP were analyzed by Open SPR instruments (Nicoya, Canada). Ligand proteins were diluted in a gradient of binding buffer (10 mM HEPES, 120 mM NaCl, 3 mM EDTA, 0.005% Tween 20, pH 7.4) and quercetin was diluted in 1% DMSO in PBS solution. After equilibrating the signal by 1% DMSO in PBS solution, imidazole and NiCl_2_ solution were added to complete the functionalization of the chip surface. After that, the ligand protein was immobilized onto the chip, and different concentrations of quercetin were added after signal smoothing with 240 s of protein-ligand binding time and 240 s of natural dissociation, and the maximum binding force on the surface was confirmed. The results were analyzed by TraceDrawer (Ridgeview Instruments ab, Sweden) and One To One.

### Pulldown assay.

All proteins used for pulldown experiments were expressed in E. coli. 6×His-ClpX protein, label-free ClpP protein, 2 mM ATPγS (Cat number 11162306001, Sigma-Aldrich) and quercetin were mixed and added to a 1.5 mL EP tube and incubated overnight at 4°C. The following day, the mixed solution was passed through the Ni-NTA column and washed with binding buffer (40 mM Tris, 200 mM NaCl, pH = 8.5), the effluent was collected and concentrated. After that, a binding buffer containing 400 mM imidazole was added to the Ni-NTA column, the effluent was collected and concentrated, and the results were finally identified by SDS-PAGE. ADEP4 (39) and DMSO were used as positive and negative controls, respectively.

### Molecular docking and dynamic simulation.

Autodock vina 1.1.2 was used for the molecular docking of quercetin and ClpP (40) to analyze the binding pattern between them. The 3D structures of ClpP (PDB ID:3V5E), quercetin (PubChem CID: 5317284), M21 (PubChem CID: 5317284), Myricetin (PubChem CID: 5281672), Dihydromyricetin (PubChem CID: 161557) and Chrysin (PubChem CID: 5281607) were obtained through the Protein Data Bank (www.rcsb.org) and PubChem database, respectively. The AutoDockTools 1.5.6 package was used to generate docking input files (41, 42). Amber 14 (43, 44) and AmberTools 15 software performed molecular dynamics simulations to correct the molecular docking results. The procedure was performed as previously described (45).

### Pneumonia model experiment.

The C57BL/6J mice (6 weeks old, female, approximately 20 g) used were provided by the Experimental Animal Center of Jilin University. There were 8 mice in each experimental group, and the S. aureus pneumonia infection model was constructed as described previously (30). Briefly, the mice were anesthetized by ether to make them upright, and then mice were intranasally inoculated with the S. aureus suspension to infect the lung tissues. The doses and routes of administration of quercetin were determined according to previous literature (46). For survival experiments, mice were infected with 2 × 10^8^ CFU/30 μL of S. aureus and treated with 100 mg/kg of quercetin administered subcutaneously 1 h and 12 h after infection. Mice were observed daily for survival and survival curves were generated. For histopathology and lung bacterial load analysis and to avoid additional mortality in mice caused by lethal doses of S. aureus, 1 × 10^8^ CFU/30 μL S. aureus was used to infect mice, followed by subcutaneous injection of 100 mg/kg quercetin for treatment at 1 h and 12 h. Mice were euthanized and treated after 24 h (the lungs of the mice at this time had the highest bacterial load to facilitate comparison). Mice were observed for lung infection and left lung tissue was taken for homogenization and CFU determination. Murine right lung tissue samples were fixed in 10% (vol/vol) formalin solution to facilitate sectioning and H&E staining.

### Statistical analysis.

Each study was repeated three times independently. Statistical comparison between groups was analyzed using Student's *t* test or one-way analysis of variance (ANOVA), and survival curve was analyzed using log-rank test. *P* < 0.05 were considered statistically significant.

### Ethical statement.

Animal experiments were strictly conducted according to the ARRIVE guidelines and the guidelines of the Animal Ethics Committee of Changchun University of Chinese Medicine.

### Data availability.

All data generated during this study are available on request from the corresponding authors.
