# Supplementary material for: Quercetin Reduces the Virulence of S. aureus by Targeting ClpP to Protect Mice from MRSA-Induced Lethal Pneumonia
Source: Microbiol Spectr. 2022 Mar 23;10(2):e02340-21. doi: 10.1128/spectrum.02340-21 (PMC9045277; doi:10.1128/spectrum.02340-21)
Supplement: SUPPLEMENTAL FILE 1 — Supplemental material. Download SPECTRUM02340-21_Supp_1_seq1.pdf, PDF file, 0.3 MB [file spectrum02340-21_supp_1_seq1.pdf]

Quercetin reduces the virulence of *S. aureus* by targeting ClpP to prevent MRSA-induced fatal pneumonia in mice

Shisong Jing[1†], Xiangri Kong[1†], Li Wang[1†], Heming Wang[3], Jiaxuan Feng[1], Lin Wei[1], Ying Meng[1], Chang Liu[1], Yishen Qu[4], Jiyu Guan[5], Haimiao Yang[2], Chi Zhang[1]*, Yicheng Zhao[1]*, Wu Song[1]*

[1.] Changchun University of Chinese Medicine, Changchun, China

[2]Affiliated Hospital to Changchun University of Chinese Medicine, Changchun 130021, China.

[3]Zhongshan Hospital Fudan University, Department of Gastroenterology and Hepatology, Shanghai, China

[4] Department of Anesthesiology, Peking University Third Hospital, Beijing, China

[5]Key Laboratory of Zoonosis, Ministry of Education, College of Veterinary Medicine, Jilin University, Changchun 130062, China.

†These authors contributed equally.

Table S1: Strains and Plasmids used in this study

Table S2: Primers used in this study

Table S3: Screening results of ClpP inhibitors among flavonoids in natural compound libraries

Figure S1. The full SDS-PAGE images of CETSA

Table S1: Strains and Plasmids used in this study

Strains or Plasmids	Source
**Strains**	
MRSA	ATCC® BAA-1717™ (USA300-HOU-MR)
MSSA	ATCC®25904 (Newman D2C)
Rosetta-pET28a–clpP	Previous study [1]
USA300-Δ*clpP*	Previous study [1]
Rosetta-pET28a-ClpP (Q47A)	This study
Rosetta-pET28a-ClpP (G33A)	This study

ATCC, American Type Culture Collection; MRSA, methicillin-resistant *Staphylococcus aureus*; MSSA, *methicillin-sensitive Staphylococcus aureus*.

Table S2: Primers used in this study

Primer name	Sequences (5´-3´)
rt-*RNAIII*-f	GCACTGAGTCCAAGGAAACTAAC
rt-*RNAIII*-r	AAGCCATCCCAACTTAATAACC
rt-*hla*-f	AAAAAACTGCTAGTTATTAGAACGAAAGG
rt-*hla*-r	GGCCAGGCTAAACCACTTTTG
rt-*spa*-f	CAGCAAACCATGCAGATGCTA
rt-*spa*-r	GCTAATGATAATCCACCAAATACAGTTG
rt-*agr*-f	GCCCATTCCTGTGCGACTTA
rt-*agr*-r	GGGCAAATGGCTCTTTGATG
rt-*lukS*-f	GAGGTGGCCTTTCCAATACAAT
rt-*lukS*-r	CCTCCTGTTGATGGACCACTATTA
rt-*psm*-f	TATCAAAAGCTTAATCGAACAATTC
rt-*psm*-r	CCCCTTCAAATAAGATGTTCATATC

rt refers to RT PCR primers.

Table S3: Screening results of ClpP inhibitors among flavonoids in natural compound libraries

Compound Name	PubChem CID	Relative inhibition rate (%) *	MIC (μg/mL) #
Quercitrin	5280343	74.3 ± 2.67	128
Nor-kurarinone	6565899	83.3 ± 0.02	2
Isobavachalcone	5281255	97.6 ± 0.26	8
Chrysin	5281607	49 ± 3.18	-
Kaempferitrin	5486199	17.5 ± 0.95	-
Cyasterone	119444	25.6 ± 2.65	-
Artemisinin	68827	4.9 ± 4.21	-
Shikonin	479503	13.4 ± 1.54	-
Isoorientin	114776	50.2 ± 1.58	-
Didymin	16760075	31.2 ± 0.59	-
Biochanin A	5280373	28.2 ± 1.38	-
Isosakuranetin	160481	28 ± 1.62	-
Kaempferol	5280863	44 ± 2.75	-
Icariin	5318997	34.6 ± 1.54	-
Baicalin	64982	27.3 ± 0.55	-
Anhydroicaritin	44259058	41.0 ± 0.81	-
Xanthotol	65090	21.6 ± 1.20	-
Baohuoside I	5488822	37.1 ± 0.59	-
Avicularin	5490064	46 ± 1.22	-
Silymarin	5213	40.6 ± 0.58	-
Iridin	5281777	14.2 ± 1.39	-
Apigenin 7,4'-dimethyl ether	5281601	18.4 ± 1.24	-
Liquiritin	503737	3.2 ± 0.97	-
Tanshinone I	114917	12.3 ± 1.41	-
Andrographolide	5318517	15.8 ± 0.72	-
Diosmetin	5281612	6.1 ± 1.68	-
Bruceine B	161496	1.6 ± 2.71	-
Orientin	5281675	61.4 ± 1.24	-
Complanatuside	5492406	12.3 ± 0.47	-

Kaempferol-3-O-glucorhamnoside	5318761	32.1 ± 0.62	-
Casticin	5315263	56.8 ± 1.22	-
Epimedin B	5748393	29.1 ± 2.01	-
Genistein	5280961	33.5 ± 0.65	-
Isoflavone	5284639	20.1 ± 0.73	-
Genistin	5281377	22.1 ± 0.63	
Naringenin	932	8.2 ± 0.24	-
Flavokawain A	5355469	2.6 ± 1.17	-
Wogonin	5281703	35.4 ± 2.11	-
Phlorizin	6072	5.3 ± 0.71	-
Tangeretin	68077	41.2 ± 1.53	-
Naringin dihydrochalcone	9894584	20.6 ± 0.51	
Pectolinarin	168849	28.3 ± 1.37	-
Genkwanin	5281617	13.7 ± 0.58	-
Ligustroflavone	10417462	26.6 ± 1.16	-
Diosmin	5281613	31.7 ± 0.92	-
Taxifolin	439533	17.2 ± 1.20	-
Silicristin	441764	9.3 ± 0.83	-
Apigenin	5280443	13.6 ± 0.52	-
Glycitin	187808	18.5 ± 1.26	-
Chrysosplenetin	5281608	52.4 ± 0.36	-
Baimaside	5282166	25.1 ± 0.69	-
Tiliroside	5320686	3.6 ± 0.17	-
Calycosin-7-glucoside	71571502	4.2 ± 0.84	-
Buddleoside	5317025	46.2 ± 1.25	-
Tectochrysin	5281954	7.1 ± 0.26	-
Epimedin A1	92043273	26.7 ± 0.81	-
Astragalin	5282102	51.3 ± 1.73	-
Typhaneoside	44566503	29.7 ± 0.72	-
Catharanthine	5458190	10.3 ± 1.62	-
Sarsasapogenin	92095	7.8 ± 0.96	-

Sakuranetin	73571	3.9 ± 1.20	-
Astilbin	119258	9.1 ± 2.73	-
Neohesperidin dihydrochalcone	30231	4.0 ± 0.71	-
Lysionotin	160921	59.1 ± 1.55	-
Pinocembrin	68071	33.7 ± 0.68	-
Bavachin	14236566	21.7 ± 1.75	-
Dehydrodiisoeugenol	5379033	5.3 ± 0.61	-
Chlorogenic acid	1794427	10.4 ± 1.12	-
Triptophenolide	173273	20.8 ± 0.61	-
Rhoifolin	5282150	46.7 ± 1.30	-
Lutein	5281243	1.4 ± 0.52	-
Farrerol	91144	33.5 ± 0.73	-
Xanthotol	65090	7.0 ± 0.21	-
Punicalagin	44584733	28.3±3.67	-
Bilobalide	73581	17.4±1.76	-
Brazilin	73384	47.2±2.57	-
Asperuloside	84298	17.4±2.31	-
Scutellarin methlester	185617	49.2±3.02	-
Rhapontigenin	5320954	42.2±2.89	-
Tigogenin	99516	12.34±4.21	-
Loureirin A	5319081	56.5±2.47	-
Loureirin B	189670	29.8±2.24	-
Fraxetin	5273569	50.3±4.62	-
6,7-Dihydroxycoumarin	5281416	19.6±3.54	-
Hematoxylin	442514	23.9±2.69	-
Alizarin	6293	56.6±5.28	-
Cryptochlorogenic acid	9798666	13.7±3.15	-
Isovanillic acid	12575	13.69±2.43	-
Prudomestin	10404353	54.2±4.67	-
luteolin-7-glucuronide	5280601	47.1±3.25	-
Vicenin-2	442664	48.2±2.26	-

Sinigrin	23682211	20.1±4.17	-
Crotonoside	65085	28.1±3.17	-
Praeruptorin A	38347607	37.8±3.15	-
Baccatin III	653666	7.2±3.26	-
Helicid	12896796	13.9±3.22	-
Clinodiside A	71571492	16.0±2.78	-
Loganin	87691	14.7±3.56	-
Hydroxysafflor yellow A	6443665	52.4±4.23	-
Purpurin	6683	11.6±2.51	-
Diosgenin glucoside	11827970	26.4±3.69	-
cannabidiol	644019	6.8±2.41	-
Paeonol	11092	26.7±4.31	-
Tetrahydropiperine	581676	24.5±3.67	-
kuwanonG	5281667	21.2±2.56	-
Abscisic acid	5375199	0.8±3.42	-
demethoxyaschantin	10926754	13.8±2.39	-
Phellodendrine chloride	59818	10.2±2.67	-
3',5-Dihydroxy-4',6,7-trimethoxyflavone	70383	55.8±2.5	-

* Relative inhibition of compounds at 64 μg/mL concentration, >65% for further validation.

MIC of compounds on MRSA bacterium USA300.

Figure S1. The full SDS-PAGE images of CETSA

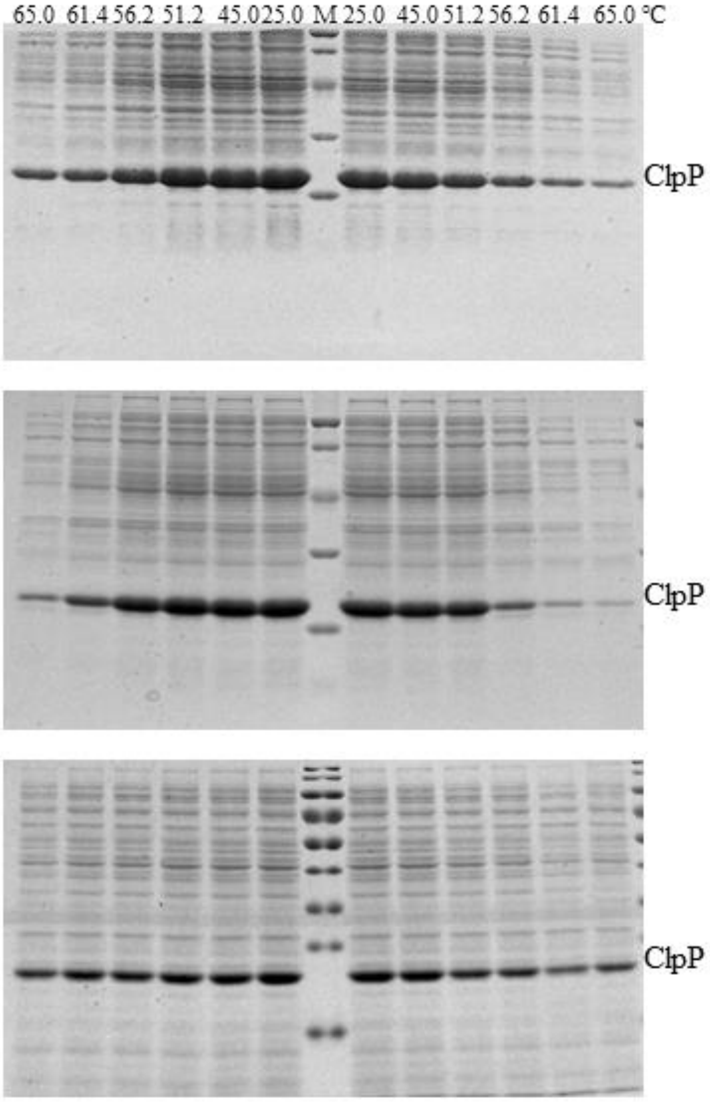

Figure S1. The full SDS-PAGE images of CETSA. Uncut SDS-PAGE images illustrated that quercetin (128 μg/mL) decreases the T_m value of ClpP protein in Rosetta-pET28a–clpP cells. Experiments were performed on three independent occasions, as indicated.

References

[1] Jing S, Wang L, Wang T, Fan L, Chen L, Xiang H, et al. Myricetin protects mice against MRSA-related lethal pneumonia by targeting ClpP. Biochemical pharmacology. 2021:114753.
